# Disease Characteristics and Treatments Associated with Outcome in Primary Angiitis of the Central Nervous System—A Multicenter Cohort Study in 163 Patients

**DOI:** 10.1002/ana.27295

**Published:** 2025-06-23

**Authors:** Anna Lena Fisse, Nadine Bonberg, Carolin Beuker, Claudia Pfeuffer, Andreas Heidenreich, Christina Krüger, Milani Deb‐Chatterji, Jana Becker, Stefan T. Gerner, Clemens Küpper, Louisa Nitsch, Roxane‐Isabelle Kestner, Lars Udo Krause, Juliane Herm, Alexander Katalinic, André Karch, Ralf Gold, Heinz Wiendl, Wolf‐Rüdiger Schäbitz, Gabor C. Petzold, Waltraud Pfeilschifter, Marius Ringelstein, Thorsten R. Doeppner, Lars Kellert, Hagen B. Huttner, Markus Kraemer, Tim Magnus, Karl Georg Haeusler, Heike Minnerup, Jens Minnerup

**Affiliations:** ^1^ Department of Neurology Ruhr University Bochum, St. Josef Hospital Bochum Germany; ^2^ Institute of Epidemiology and Social Medicine, University of Muenster Muenster Germany; ^3^ Department of Neurology University of Muenster Münster Germany; ^4^ Institute of Social Medicine and Epidemiology, University of Luebeck Luebeck Germany; ^5^ Department of Neurology University Hospital Hamburg‐Eppendorf Hamburg Germany; ^6^ Department of Neurology University Hospital Schleswig‐Holstein Kiel Germany; ^7^ Department of Neurology Alfried Krupp Hospital Essen Germany; ^8^ Department of Neurology University Hospital Erlangen Erlangen Germany; ^9^ Department of Neurology kbo‐Isar‐Amper‐Hospital Munich East Munich Germany; ^10^ Department of Neurology University Hospital Bonn Bonn Germany; ^11^ Department of Neurology University Hospital Frankfurt Frankfurt am Main Germany; ^12^ Department of General Pharmacology and Toxicology Goethe University Frankfurt am Main Germany; ^13^ Department of Neurology Klinikum Osnabrück Osnabrück Germany; ^14^ Department of Neurology Charité—Universitätsmedizin Berlin Berlin Germany; ^15^ Department of Neurology and Neurophysiology Medical Center, University of Freiburg Freiburg Germany; ^16^ Department of Neurology Evangelisches Klinikum Bethel, University Hospital OWL of the University Bielefeld, Campus Bielefeld‐Bethel Bielefeld Germany; ^17^ Department of Neurology Klinikum Lüneburg Lüneburg Germany; ^18^ Department of Neurology Medical Faculty and University Hospital Düsseldorf, Heinrich Heine University Düsseldorf Düsseldorf Germany; ^19^ Department of Neurology Centre for Neurology and Neuropsychiatry, LVR‐Klinikum, Heinrich‐Heine‐University Düsseldorf Düsseldorf Germany; ^20^ Department of Neurology Justus Liebig University, University Hospital Giessen and Marburg Giessen Germany; ^21^ Center for Mind, Brain and Behavior (CMBB), Philipps University Marburg, University Hospital Giessen and Marburg Marburg Germany; ^22^ Department of Neurology University of Goettingen Medical School Goettingen Germany; ^23^ Department of Neurology LMU University Hospital, LMU Munich Munich Germany; ^24^ Department of Neurology University Hospital Carl Gustav Carus, Technische Universität Dresden Dresden Germany; ^25^ Department of Neurology Universitätsklinikum Würzburg (UKW) Würzburg Germany; ^26^ Department of Neurology Universitätsklinikum Ulm Ulm Germany; ^27^ Department of Neurology University Hospital Schleswig Holstein, Campus Lübeck, and University of Lübeck Lübeck Germany

## Abstract

**Objective:**

The aim was to determine patient, disease, and treatment characteristics associated with outcome in patients with primary angiitis of the central nervous system (PACNS) in a large multicenter German cohort.

**Methods:**

In a retrospective, observational cohort study, we analyzed 163 adult patients who met the diagnostic criteria for PACNS. Data were collected from January 2004 to December 2018 in 13 tertiary care centers in Germany. Survival, recurrence‐free survival and long‐term global disability were assessed.

**Results:**

Of 163 patients with PACNS (median [interquartile range (IQR)] age 48 [39–59.5] years; 73 [45%] women), 29 (18%) died, 84 (52%) had a poor outcome (modified Rankin scale [mRS] 3–6 at last follow‐up), and 82 (50%) patients relapsed. Poorer survival was associated with patient age (hazard ratio [HR], 1.96 [95% confidence interval (CI), 1.41–2.74] per 10 years), longer time between initial manifestation and diagnosis (HR, 1.01 [95% CI, 1.00–1.01] per month), and necrotizing subtype (HR, 10.2 [95% CI, 2.18–48.2]). Long‐term disability was associated with older age (odds ratio [OR], 1.40 [95% CI, 1.07–1.86] per 10 years), and worse mRS score at diagnosis (OR, 4.43 [95% CI, 1.97–10.4]). Patients treated with cyclophosphamide alone or in combination with steroids had a lower incidence of relapse than untreated patients (HR, 0.44 [95% CI, 0.22–0.86]; HR, 0.47 [95% CI, 0.24–0.92]).

**Interpretation:**

In patients with PACNS, the long‐term outcome depends on several patient and disease characteristics. Our results favor treatment with cyclophosphamide alone or in combination with steroids, because this was associated with a reduction in the relapse rate. ANN NEUROL 2025;98:883–893

Primary angiitis of the central nervous system (PACNS) is a rare autoimmune disease that affects exclusively the central nervous system (CNS).^1^ Patients present with a highly variable and non‐specific spectrum of symptoms depending on the part of the CNS affected. Clinical manifestations at diagnosis include focal neurologic symptoms because of recurrent episodes of ischemia or hemorrhage, headache, as well as cognitive and affective abnormalities associated with encephalopathy. The disease is characterized by inflammation and subsequent destruction of CNS arteries.^2^ The diagnosis of PACNS requires evidence of vessel wall changes demonstrated by angiography or biopsy.^3^ However, the multiple symptoms of PACNS and the non‐specificity of available diagnostic modalities make it challenging to correctly diagnose and differentiate PACNS from other diseases. Additionally, PACNS represents a spectrum of disorders that affect cerebral arteries of different sizes and are characterized by different clinical and laboratory findings.^4^, ^5^ Careful exclusion of other diseases is essential, as unwarranted immunosuppression can have devastating consequences. In this regard, the most common clinical and radiological mimic of PACNS is reversible cerebral vasoconstriction syndrome (RCVS).^6^ However, because the validation of specific features distinguishing PACNS from RCVS,^6^, ^7^ intracranial atherosclerosis (ICAD) has emerged as the most critical differential diagnosis, particularly in patients with multifocal involvement of large and medium‐sized vessels.

To date, no randomized clinical trials have been conducted for the treatment of patients with PACNS. Current treatment recommendations are based on observational studies or derived from the treatment of systemic vasculitides.^8^ The most commonly used therapeutic agents are corticosteroids for initial stabilization, followed by various immunosuppressive maintenance therapies.^9^ However, data on the long‐term course of the disease, predictive patient or disease factors, and optimal treatment strategies are very limited.

To address this need, we assembled a large multicenter cohort of patients with PACNS to analyze the association of clinical, imaging, and laboratory characteristics with treatment regimens and outcomes.

## Methods

### Standard Protocol Approvals, Registrations, and Patient Consents

The study was conducted according to the Declaration of Helsinki and approved by the local ethics committee of all participating centers (reference number 2016_218_f_S). All patients were recruited consecutively. Written informed consent was obtained from all patients or legal representatives.

### Study Oversight

We conducted a multicenter historical cohort study of patients with PACNS age 18 years or older who had been examined in one of 13 tertiary care centers in Germany over 15 years from January 2004 to December 2018. Patients who fulfilled both the original diagnostic criteria established by Calabrese and Mallek^3^ in 1988, as well as the more recent criteria proposed by Birnbaum and Hellmann,^10^ were included in the study. The Calabrese criteria require (1) the presence of an acquired neurological or psychiatric deficit of otherwise unexplained origin, (2) classic angiographic or pathological features of CNS angiitis, and (3) the absence of systemic vasculitis or other disorders that could cause or mimic the angiographic or pathological features of the disease. The 2009 criteria by Birnbaum and Hellmann^10^ further differentiate between (1) patients with a definitive PACNS diagnosis confirmed by tissue biopsy demonstrating vasculitis and (2) patients with a probable PACNS diagnosis who lack histological confirmation, but exhibit highly suggestive angiographic findings, along with abnormal magnetic resonance imaging (MRI) findings and a cerebrospinal fluid (CSF) profile consistent with PACNS. All included cases were confirmed by biopsy or angiogram. Patients with both a positive brain biopsy and a positive angiogram were classified as “biopsy confirmed”. The term “angiogram confirmed” refers to any type of angiography, that is, digital subtraction, magnetic resonance, or computed tomography (CT) angiography. In angiographically confirmed cases, we included only those that closely adhered to the defined angiographic pattern, characterized by (1) alternating areas of smooth‐walled, segmental narrowing and dilation of the cerebral arteries, (2) arterial occlusion affecting multiple cerebral vessels, and (3) absence of atherosclerosis in proximal vessels or other known abnormalities.^11^

Data on demographics, medical history, cerebrovascular risk factors, PACNS diagnostic characteristics (symptoms, imaging characteristics, MRI and angiogram scans, biopsy, and CSF findings, as well as laboratory data), and immunotherapy were extracted from patients medical records and institutional databases at each institution. The following clinical manifestations were included under the umbrella term focal neurological symptoms at diagnosis: paresis, aphasia, ataxia, hypesthesia, and/or dysarthria. The start and duration of all therapeutic regimens were recorded. Immunotherapies were classified into the following groups: (1) no treatment, (2) steroid monotherapy, (3) cyclophosphamide monotherapy, (4) steroid plus cyclophosphamide, (5) steroid plus other immune therapy, and (6) other immune therapy. Other immune therapies included azathioprine, mycophenolate mofetil, rituximab, methotrexate, plasma exchange, intravenous immunoglobulins, ciclosporin, tacrolimus, leflunomide, infliximab, tocilizumab, and etanercept.

### Outcomes

We obtained follow‐up data on mortality, clinical symptoms, functional outcomes, relapses, and maintenance therapy through semiquantitative telephone interviews. In cases of missing contact information, a local registry office inquiry was made to complete the outcome assessment. The main clinical outcomes were as follows: (1) survival, (2) relapse‐free survival, and (3) global disability (modified Rankin scale [mRS] score >2) at the last follow‐up visit. Relapse was defined as new symptoms and/or worsening of PACNS symptoms and/or new lesions on subsequent MRI scans and/or worsening of pre‐existing neuroimaging changes consistent with PACNS activity. Subjects were followed until their death or the last follow‐up visit.

### Statistical Analysis

Statistical analyses were performed using R 4.0.0 and 4.1.3 (www.r-project.org). Data were presented as (relative) frequencies and as medians (with range and interquartile range [IQR]) as appropriate. Group comparisons were performed with the Wilcoxon rank sum test for continuous or Fisher's exact test for categorical variables.

For survival analysis, the R‐packages survival^12^, ^13^ and survminer^14^ were used. Survival and relapse‐free survival were estimated with the Kaplan–Meier method. For the figures, we used the R packages ggplot^15^ and easyalluvial.^16^

Four Cox proportional hazards models were defined to estimate the effect of (1) clinical variables, (2) clinical and imaging variables (3), CSF characteristics, and (4) biopsy characteristics on survival and relapse‐free survival, respectively. Analogously, logistic regression models were used to assess the relationship between (1)–(4) and a poor functional outcome (mRS >2). All models were adjusted for age at diagnosis, method of diagnosis, sex, mRS at diagnosis, and time from primary manifestation to diagnosis. Hazard ratios (HR) and odds ratios (OR) were reported with 95% confidence intervals (95% CI).

To assess the effect of treatments on the risk of relapses, we used a time‐dependent extended Cox model (counting process model) according to Andersen and Gill with treatment as a time‐dependent covariate. To adjust for the severity of the disease, we calculated the number of previous relapses at each time point. Additionally, the model was adjusted for age at diagnosis, sex, mRS at diagnosis, time from primary manifestation to diagnosis, and leukoencephalopathy.

## Results

In total, 163 patients diagnosed with PACNS were included in the study. Among these cases, 105 (64%) were biopsy confirmed and 58 (36%) angiogram confirmed.

### Baseline Characteristics

The characteristics of the entire study population as well as stratified by mode of diagnosis, are shown in Table 1. The median age at the time of diagnosis was 48 years (range: 15–85), and 73 patients (45%) were women. The median time from the onset of symptoms to diagnosis was 3 months (range: 0–355) in cases diagnosed by biopsy and 2 months (range: 0–123 months) in those diagnosed by angiogram. The median mRS at diagnosis was 3 (range: 0–5) and did not differ between biopsy‐ and angiogram‐confirmed cases. A broad spectrum of clinical symptoms was present at the time of diagnosis. Angiogram‐confirmed compared to biopsy‐confirmed cases had more hemiparesis (54% vs 37%, *p* = 0.05) and fewer seizures (16% vs 48%, *p* < 0.001). Cerebrovascular risk factors, including arterial hypertension and smoking were reported more frequently for angiogram‐confirmed cases compared to biopsy‐confirmed cases (50% vs 32%, *p* = 0.03; 47% vs 23%, *p* < 0.01).

**TABLE 1:** Patient and Disease Characteristics at Diagnosis

	All patients n = 163	Biopsy‐confirmed n = 105	Angiogram‐confirmed n = 58	*p*
Demographics				
Women, n (%)	73/163 (45)	50/105 (48)	23/58 (40)	0.41
Median age at diagnosis, yr (range, IQR)	48 (15–85, IQR: 39–59.5)	49 (15–83, IQR: 39–63)	48 (20–85, IQR: 39.5–58)	0.64
Clinical manifestations at diagnosis, n (%)				
Headache	78/162 (48)	48/105 (46)	30/57 (53)	0.42
Cognitive impairment	84/162 (52)	58/104 (56)	26/58 (45)	0.19
Hemiparesis	70/162 (43)	39/105 (37)	31/57 (54)	**0.05**
Paraparesis	8/162 (5)	5/105 (5)	3/57 (5)	1.00
Tetraparesis	11/162 (7)	8/105 (8)	3/57 (5)	0.75
Aphasia	46/162 (28)	32/105 (30)	14/57 (25)	0.47
Ataxia	38/162 (23)	27/105 (26)	11/57 (19)	0.44
Hemihypesthesia	56/162 (35)	36/105 (34)	20/57 (35)	1.00
Seizure	59/162 (36)	50/105 (48)	9/57 (16)	**<0.001**
Extrapyramidal symptoms	8/162 (5)	6/105 (6)	2/57 (4)	0.71
Impaired vision	52/162 (32)	32/105 (30)	20/57 (35)	0.60
Dysarthria	32/162 (20)	18/105 (17)	14/57 (25)	0.30
Vertigo	31/162 (19)	19/105 (18)	12/57 (21)	0.68
B symptoms^a^	8/162 (5)	5/105 (5)	3/57 (5)	1.00
Comorbidities, n (%)				
Atrial fibrillation	8/160 (5)	7/102 (7)	1/58 (2)	0.26
Arterial hypertension	62/161 (39)	33/103 (32)	29/58 (50)	**0.03**
Diabetes mellitus	24/160 (15)	17/102 (17)	7/58 (12)	0.50
Stenosis/occlusion of the ICA	4/160 (2)	2/102 (2)	2/58 (3)	0.62
Peripheral arterial disease	4/160 (2)	2/102 (2)	2/58 (3)	0.62
Smoking	50/160 (31)	23/102 (23)	27/58 (47)	**<0.01**
Coronary heart disease	6/159 (4)	3/102 (3)	3/57 (5)	0.67
Dyslipidemia	35/160 (22)	19/102 (19)	16/58 (28)	0.23
Disease characteristics, n (%)				
No. of deaths	29/163 (18)	17/105 (16)	12/58 (21)	0.52
No. of patients with relapses	82/163 (50)	48/105 (46)	34/58 (59)	0.14
Median time from primary manifestation to diagnosis, mo (range, IQR)	3 (0–355, IQR: 1–14.5)	3 (0–355, IQR: 1–19)	2 (0–123, IQR: 1–5)	0.09
Median mRS at diagnosis, (range, IQR)	3 (0–5, IQR: 1.5–3)	3 (0–5, IQR: 1–3)	3 (0–5, IQR: 2–3)	0.94
Rapid progressive course^b^	13/162 (8)	9/104 (9)	4/58 (7)	0.77
Median follow‐up time, mo (range, IQR)	42 (1–263, IQR: 17–77)	44 (1–263, IQR: 17–80)	40 (1–203, IQR: 14.75–76.75)	0.93

*Note:* Values in bold are statistically significant *p* < 0.05.

^a^ B symptoms: fever, night sweats, weight loss >10% of total body weight within 6 mo.

^b^ Rapid progression: symptom recurrence or marked worsening within 7 days.

ICA = internal carotid artery; IQR = interquartile range; mo = month; mRS = modified Rankin scale; yr = year.

Cerebral imaging was performed in all patients (Table 2). MRI was performed in 161 (99%) and CT was performed in 65 (40%) patients. Spinal MRI was performed in 30 (18%) of all patients. Both acute and old infarctions were found more frequently on angiogram‐confirmed compared to biopsy‐confirmed patients (67% vs 28%, *p* < 0.001; 55% vs 26%, *p* < 0.001), whereas microhemorrhages (18% vs 5%, *p* = 0.02), and mass lesions (35% vs 3%, *p* < 0.001) were significantly more frequent in biopsy‐confirmed cases. In 20 (32%) of the 62 biopsy‐confirmed patients, magnetic resonance angiography (MRA) showed classical features of cerebral vasculitis. Of 39 cases that were confirmed by angiogram and MRA was performed, it was positive in 32 (82%) cases.

**TABLE 2:** Imaging, Biopsy, and CSF Characteristics at Diagnosis

	All patients n = 163	Biopsy‐confirmed n = 105	Angiogram‐confirmed n = 58	*p*
Cerebral MRI performed, n (%)	161/163 (99)	103/105 (98)	58/58 (100)	0.54
Spinal MRI performed, n (%)	30/163 (18)	24/105 (23)	6/58 (10)	0.06
CT performed, n (%)	65/163 (40)	36/105 (34)	29/58 (50)	0.07
Imaging characteristics, n (%)				
Acute infarction at diagnosis	68/162 (42)	29/104 (28)	39/58 (67)	**<0.001**
Old infarctions	59/162 (36)	27/104 (26)	32/58 (55)	**<0.001**
Leukoencephalopathy	46/162 (28)	34/104 (33)	12/58 (21)	0.15
Acute hemorrhage	24/162 (15)	15/104 (14)	9/58 (16)	0.82
Microhemorrhage	22/161 (14)	19/103 (18)	3/58 (5)	**0.02**
Gadolinium‐enhanced parenchymal lesions	62/122 (51)	48/85 (56)	14/37 (38)	0.08
Leptomeningeal enhancement	17/121 (14)	14/84 (17)	3/37 (8)	0.27
Mass lesion	38/161 (24)	36/103 (35)	2/58 (3)	**<0.001**
Spinal involvement	12/28 (43)	11/22 (50)	1/6 (17)	0.20
Pathological MR‐angiography	52/101 (51)	20/62 (32)	32/39 (82)	**<0.001**
Biopsy characteristics, n (%)				
Biopsy performed	120/163 (74)	105/105 (100)	15/58 (26)	**<0.001**
Pathological biopsy findings, n (%)	105/120 (88)	105/105 (100)	0/15 (0)	**<0.001**
ABRA, n (%) ABRA	13/120 (11)	13/105 (12)	0/15 (0)	0.37
Granulomatous, n (%)	12/120 (10)	12/105 (11)	0/15 (0)	0.36
Lymphocytic, n (%)	55/120 (46)	55/105 (52)	0/15 (0)	**<0.001**
Necrotizing, n (%)	16/120 (13)	16/105 (15)	0/15 (0)	0.22
Coronary heart disease	6/159 (4)	3/102 (3)	3/57 (5)	0.67
Dyslipidemia	35/160 (22)	19/102 (19)	16/58 (28)	0.23
CSF characteristics				
CSF analysis performed, n (%)	154/159 (97)	96/101 (95)	58/58 (100)	0.16
Cell count >5/μL, n (%)	99/147 (67)	41/89 (46)	58/58 (100)	**<0.001**
Protein concentration >450mg/L, n (%)	96/126 (76)	60/80 (75)	36/46 (78)	0.83
Median cell count (range, IQR), /μL	10 (0–3,024, IQR: 36.25)	4.5 (0–3,024, IQR: 17)	26 (6–2,184, IQR: 17)	0.41
Median protein concentration (range, IQR), mg/L	667 (72.6–9,779, IQR: 369)	651 (72.6–9,779, IQR: 317)	681.5 (92–6,968, IQR: 317)	0.61

The term “angiogram‐confirmed” refers to any type of angiography, that is, digital subtraction, MR, and CT angiography.

ABRA = amyloid‐beta‐related angiitis; CSF = cerebrospinal fluid; CT = computed tomography; IQR = interquartile range; MR = magnetic resonance; MRI = magnetic resonance imaging.

Histological samples of brain biopsies were obtained in 120 (74%) patients and showed vasculitis in 105 (88%) cases (Table 2). In 15 (12%) cases without evidence of PACNS on biopsy, vasculitis characteristics were found on cerebral angiogram. In biopsy‐positive patients, a lymphocytic pattern was found in 55 (52%) patients, a granulomatous pattern in 12 (11%) patients, and a necrotizing pattern in 16 (15%) patients. Amyloid‐beta‐associated angiitis (ABRA) was detected in 13 (12%) cases of biopsy samples. Among the 105 biopsy‐positive patients, 14 patients had overlaps of pathologies. CSF examinations were performed on 154 (97%) patients (Table 2). An elevated cell count (>5 cells/μL) was present in 99 (67%) of all cases, in 41 (46%) cases of those diagnosed by biopsy and 58 (100%) of those diagnosed by angiogram. Pathological results in terms of an increased protein concentration (>450mg/L) were apparent in 60 (75%) cases confirmed by biopsy and 36 (78%) by angiogram.

### Survival, Relapse‐Free Survival, and Long‐Term Disability

**FIGURE 1:**
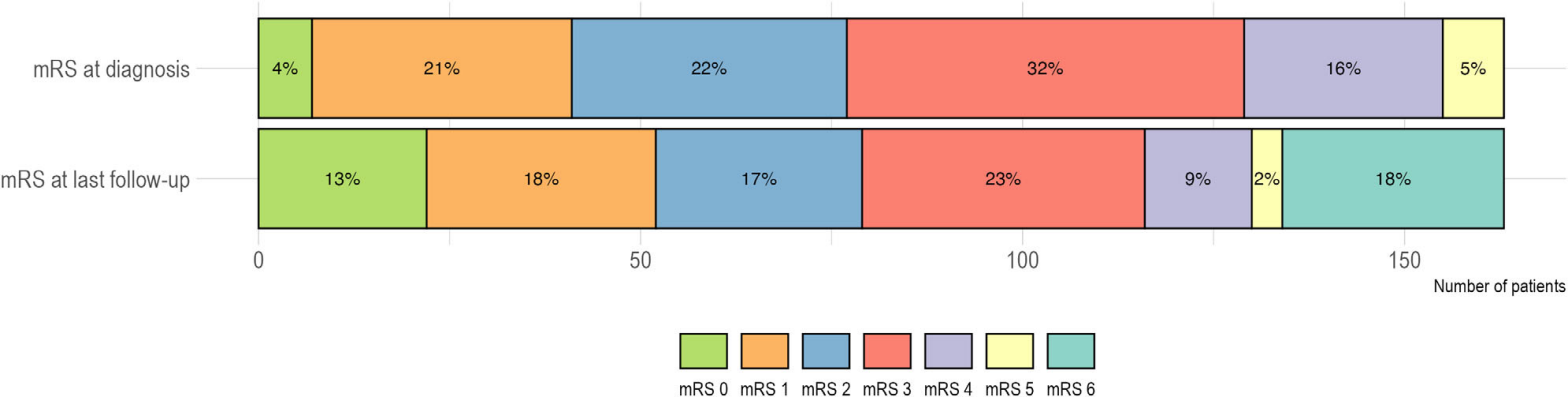
Distribution of functional outcome in all patients at diagnosis and the last follow‐up using the modified Rankin scale (mRS). An mRS of 0 indicates that there are no symptoms; mRS 1, no significant disability; mRS 2, slight disability; mRS 3, moderate disability; mRS 4, moderately severe disability; mRS 5, severe disability; and mRS 6, death.

**FIGURE 2:**
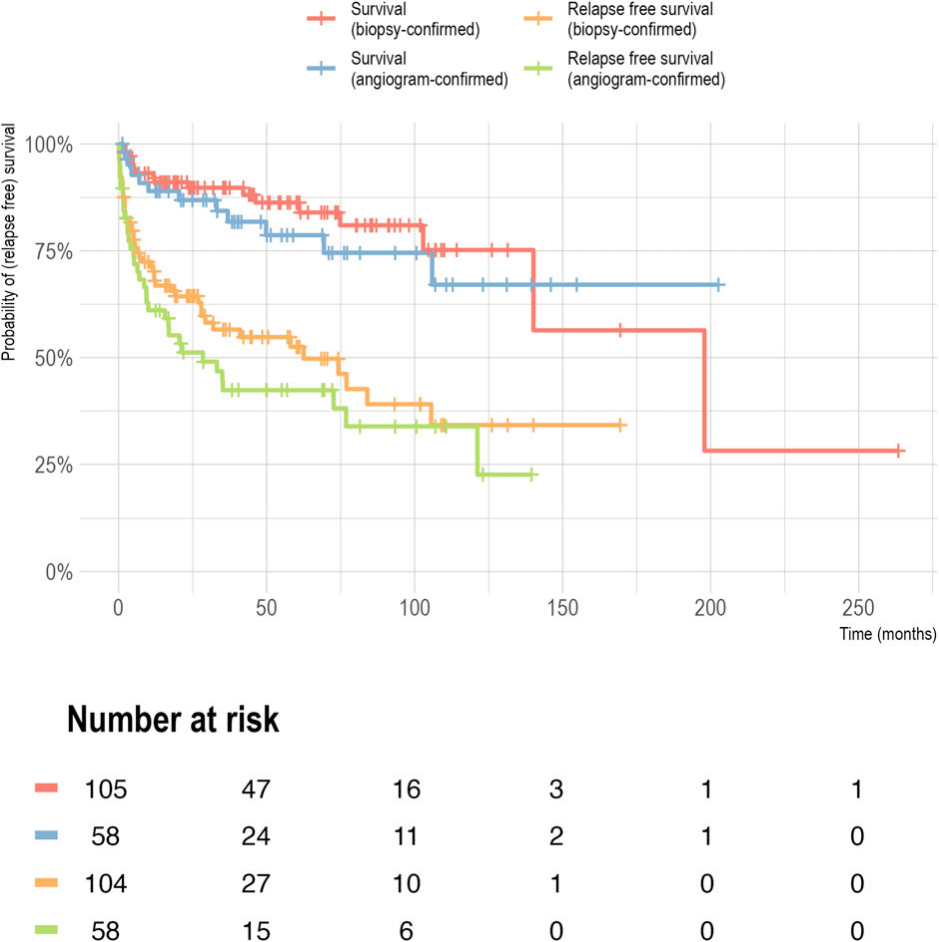
Kaplan–Meier survival and relapse‐free survival curves in patients with primary angiitis of the central nervous system (PACNS). Probability of survival and relapse‐free survival in biopsy‐confirmed cases compared to angiogram‐confirmed cases.

Patients were followed up to their death or their last visit up to December 2018 (Table 1). Median time under observation for all patients was 42 months (range: 1–263) and median time to relapse in those with a relapse was 18 months (IQR: 4–55 months). Relapses occurred in 82 (50%) of all patients and 13 (8%) patients with a rapidly progressive disease course, defined as the recurrence of symptoms (eg, new infarcts or hemorrhage) or distinct worsening of symptoms within 7 days of the initial event. The distribution of mRS at diagnosis and last follow‐up visit is shown in Figure 1. The proportion of patients with a good outcome (mRS, 0–2) showed no significant differences between the time of diagnosis and the last follow‐up. The proportion of patients with moderate to severe disability (mRS, 3–5) decreased (34% vs 53%). Overall, there were 29 (18%) deaths and 84 patients (52%) had poor outcomes indicated by mRS 3 to 6 at the last follow‐up. Survival and relapse‐free survival were comparable among angiogram‐ and biopsy‐confirmed cases (Fig 2).

### Patient and Disease Characteristics as Predictors of Outcome

Patient and disease characteristics at the time of diagnosis that were associated with survival, relapse‐free survival, and long‐term disability are shown in Table 3. Overall survival and relapse‐free survival time did not differ significantly between biopsy‐confirmed and angiogram‐confirmed patients (Table 3). Increasing age was associated with higher mortality (HR, 1.96 [95% CI, 1.41–2.74] per 10 years of age; *p* < 0.001) and long‐term disability (OR, 1.40 [95% CI, 1.07–1.86] per 10 years of age; *p* = 0.02). Patients with high disability scores (mRS >2) at diagnosis were more likely to suffer long‐term disability (OR, 4.43 [95% CI, 1.97–10.4]; *p* < 0.001). A longer time from primary manifestation to diagnosis was associated with higher mortality (HR, 1.01 [95% CI, 1.00–1.01] per month; *p* = 0.01). Imaging findings and CSF characteristics at diagnosis were not associated with survival, relapses, or long‐term disability (Table 3). Regarding the biopsy findings, there were higher mortality rates in patients with necrotizing histological patterns (HR, 10.2 [95% CI, 2.18–48.2]; *p* < 0.01) compared to other histopathological subtypes. Relapse‐free survival and long‐term disability were not associated with different histopathological subtypes (Table 3).

**TABLE 3:** Patient and Disease Characteristics Associated with Outcome

	Survival (Cox regression)					Relapse (Cox regression)					Disability (logistic regression)				
	n	Events	HR	95% CI	*p*	n	Events	HR	95% CI	*p*	n	Events	OR	95% CI	*p*
Model 1, clinical manifestations at diagnosis															
Age at diagnosis (per 10 yr)	155	28	1.96	1.41–2.74	**<0.001**	154	77	1.06	0.90–1.25	0.50	155	80	1.40	1.07–1.86	**0.02**
Time from primary manifestation to diagnosis (per mo)	155	28	1.01	1.00–1.01	**0.01**	154	77	1.00	1.00–1.01	0.96	155	80	1.01	1.00–1.03	0.07
Headache without focal neurological symptoms	76	11	0.74	0.31–1.81	0.52	76	42	1.24	0.77–2.00	0.38	76	35	0.86	0.41–1.84	0.70
Cognitive impairment	81	21	1.28	0.43–3.75	0.66	81	40	0.92	0.56–1.50	0.74	81	50	1.23	0.55–2.71	0.60
Focal neurological symptoms^a^	105	24	1.15	0.35–3.73	0.82	105	50	0.66	0.39–1.13	0.13	105	64	1.74	0.74–4.09	0.20
mRS at diagnosis >2	83	23	2.86	0.87–9.40	0.08	82	42	1.38	0.81–2.34	0.24	83	59	4.43	1.97–10.4	**<0.001**
Cardiovascular risk factors ≥2^b^	50	9	1.13	0.49–2.61	0.78	50	25	0.96	0.58–1.58	0.87	50	29	1.45	0.64–3.33	0.38
Model 2, imaging findings at diagnosis															
Acute infarct at diagnosis	66	12	1.34	0.51–3.57	0.55	66	38	1.54	0.90–2.64	0.12	66	36	1.18	0.50–2.80	0.71
Acute hemorrhage	24	5	1.32	0.42–4.10	0.64	24	11	0.85	0.43–1.67	0.63	24	14	1.09	0.38–3.20	0.88
Leukoencephalopathy	44	15	1.75	0.66–4.64	0.27	44	24	1.42	0.82–2.44	0.21	44	30	1.73	0.70–4.35	0.24
Model 3, CSF characteristics															
Cell count >5/μL	82	16	1.03	0.29–3.59	0.97	82	45	1.33	0.60–2.95	0.48	82	45	1.41	0.46–4.38	0.55
Protein concentration >450mg/L	95	19	2.79	0.53–14.8	0.23	95	44	1.59	0.77–3.28	0.21	95	50	0.68	0.23–1.96	0.48
Model 4, biopsy characteristics															
ABRA	13	6	6.98	0.93–52.4	0.06	13	6	0.81	0.28–2.39	0.71	13	7	0.79	0.15–4.10	0.78
Granulomatous	12	2	0.40	0.05–3.13	0.38	12	6	1.61	0.60–4.33	0.35	12	5	0.41	0.08–1.82	0.26
Necrotizing	16	5	10.2	2.18–48.2	**<0.01**	16	5	0.97	0.36–2.64	0.96	16	9	0.90	0.23–3.60	0.88
Lymphocytic	55	9	4.27	0.96–19.0	0.06	55	26	1.18	0.59–2.36	0.64	55	28	1.11	0.39–3.19	0.85

Model 1: adjusted for age at diagnosis, sex, method of diagnosis, and time from primary manifestation to diagnosis; model 2: adjusted for age at diagnosis, sex, method of diagnosis, and time from primary manifestation to diagnosis, and clinical manifestations at diagnosis covered in model 1; model 3: adjusted for age at diagnosis, sex, mRS at diagnosis, time from primary manifestation to diagnosis and method of diagnosis; model 4: biopsy‐positive patients only, adjusted for age at diagnosis, sex, mRS at diagnosis, and time from primary manifestation to diagnosis.

^a^ Focal neurological symptoms: paresis, aphasia, ataxia, hypesthesia, and/or dysarthria.

^b^ Atrial fibrillation, arterial hypertension, diabetes mellitus, internal carotid artery stenosis/occlusion, peripheral arterial disease, smoking, coronary heart disease, and dyslipidemia.

ABRA = amyloid‐beta‐related angiitis; CI = confidence interval; CSF = cerebrospinal fluid; HR = hazard ratio; mo = month; mRS = modified Ranking scale; OR = odds ratio; yr = year.

### Therapy

**FIGURE 3:**
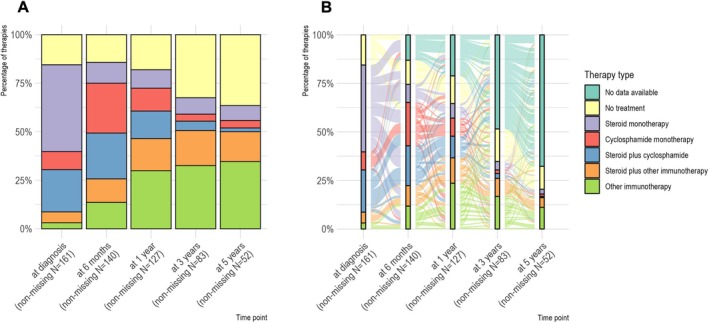
Overview of applied treatment combinations and transitions between therapies during follow‐up. Treatments are indicated by numbers and colors. (A) The percentages of different therapies for each time point of follow‐up are shown in their respective boxes. (B) Transitions between therapeutic regimens during follow‐up are shown. Individual complex combinations of therapies were used during the course of the disease. No data available indicates lost‐to‐follow‐up within the entire follow‐up period of a maximum of 5 years.

In total, 647 years of treatment were analyzed. Steroid monotherapy was applied for a total of 74 patient years, cyclophosphamide monotherapy for 50 patient years, the combination of cyclophosphamide and steroids for 54 patient years, and steroids plus other immunotherapies for 94 patient years. Other immunotherapies were applied for 184 patient years. The most common induction regimens were steroid monotherapy (45%) and the combination of steroids plus cyclophosphamide (22%) (Fig 3A). At 6 months, the most common regimens were cyclophosphamide monotherapy (26%) and the combination of cyclophosphamide plus steroids (24%). At 5 years, the majority of patients had no immunotherapy (37%), followed by immune therapies other than steroids or cyclophosphamide (35%). Figure 3B illustrates the frequency of initial therapies and the transitions between these treatment strategies during follow‐up. Table 4 provides adjusted HRs for the effect of different therapeutic regimens on relapse frequency adjusted for clinical, imaging, and biopsy characteristics. Notably, relapses occurred less frequently among patients treated with cyclophosphamide monotherapy (HR, 0.44 [95% CI, 0.22–0.86]; *p* = 0.02) or the combination of steroids and cyclophosphamide (HR, 0.47 [95% CI, 0.24–0.92]; *p* = 0.03) compared to untreated patients. There were no differences in relapse frequency between the biopsy‐confirmed and angiogram‐confirmed cases under the different immunotherapies.

**TABLE 4:** Therapies Associated with the Occurrence of Relapses

Reference: no therapy	Biopsy‐confirmed and angiogram‐confirmed			Biopsy‐confirmed			Angiogram‐confirmed		
	HR	95% CI	*p*	HR	95% CI	*p*	HR	95% CI	*p*
Steroid monotherapy	0.92	0.49–1.75	0.81	1.22	0.56–2.65	0.62	0.66	0.24–1.86	0.44
Cyclophosphamide monotherapy	0.44	0.22–0.86	**0.02**	0.53	0.23–1.22	0.13	0.33	0.09–1.18	0.09
Steroid plus cyclophosphamide	0.47	0.24–0.92	**0.03**	0.48	0.20–1.18	0.11	0.60	0.20–1.82	0.36
Steroid plus other immunotherapy	0.69	0.38–1.27	0.23	0.77	0.38–1.56	0.46	0.47	0.10–2.14	0.33
Other immunotherapy	0.89	0.56–1.44	0.65	0.93	0.50–1.71	0.81	1.14	0.53–2.43	0.74

Results from extended time‐dependent Cox models adjusted for age at diagnosis, sex, mRS at diagnosis, time from primary manifestation to diagnosis, leukoencephalopathy, and the number of previous relapses.

CI = confidence interval; HR = hazard ratio; mRS = modified Rankin scale.

### Discussion

This nationwide cohort of 163 patients represents the world's largest cohort of biopsy‐confirmed adult patients with PACNS with long‐term follow‐up. Our study improves the knowledge about the clinical characteristics, outcome, and treatment of this rare disease. Our analysis has the following main findings: (1) half of the patients had relapses with a median time to relapse of 18 months; (2) approximately half of the patients have a long‐term unfavorable functional outcome (mRS, 3–6); (3) patient age, mRS at diagnosis, time from primary manifestation to diagnosis, and the presence of a necrotizing pattern were significantly associated with a worse outcome; and (4) treatment with cyclophosphamide alone or in combination with steroids was associated with a considerably lower risk of relapses.

In line with previous studies, our data support the differentiation of PACNS patients into biopsy‐confirmed and angiogram‐confirmed cases based on the size of the affected vessels (small vs large vessels). As demonstrated in recent studies, both old and acute infarcts were less common in biopsy‐confirmed cases within our cohort.^5^, ^17^ However, in contrast to prior findings, we observed a significantly higher incidence of mass lesions and microhemorrhages in biopsy‐confirmed cases. It should be noted that these differences between the two subtypes may also be biased by the diagnostic pathway. For instance, it is conceivable that the presence of multiple ischemic lesions of different ages along with positive angiographic evidence strengthened the diagnosis of vasculitis and, therefore, rendered a biopsy unnecessary. Conversely, the detection of a mass lesion may have necessitated the performance of a biopsy because of differential diagnostic considerations.

Regarding the association between the histopathological pattern and treatment outcomes, Salvarani et al^18^ reported that the lymphocytic pattern was associated with lower mortality and disability, although these patients were less likely to achieve long‐term remission compared with the granulomatous and necrotizing patterns. In our study, we found no relevant impact of the lymphocytic pattern on outcome, but we did find that a necrotizing pattern was associated with a decrease in overall survival. These discrepancies may be because of differences in the number of patients with a lymphocytic pattern (n = 55 in our cohort compared to n = 17 in Salvarani et al^18^) and the duration of follow‐up.

Glucocorticoids in combination with cyclophosphamide are the current standard of care for the treatment of PACNS.^4^ However, in the absence of randomized clinical trials of PACNS treatment, these treatment recommendations are based on small observational studies and are guided by the corresponding therapeutic strategies of systemic vasculitides. Our study is the largest to show that cyclophosphamide, with or without steroid combination, is associated with less frequent relapses. This finding is consistent with the results of previous smaller observational studies.^19^, ^20^

We observed favorable long‐term outcomes in only approximately half of the patients. On the contrary, the Mayo Clinic cohort (median follow‐up time 12 months) and the French Cohort of Primary Cerebral Vasculitis (COVAC) cohort (median follow‐up 57 months) had a higher percentage of patients with good functional status (mRS ≤2) at the last follow‐up (80–85% and 56%, respectively). The discrepancies in the results may be explained by the higher frequency of relapses in our cohort. In comparison, long‐term relapse‐free remission rates of only 21 to 66% have been reported in recent studies.^18^, ^19^ These findings underscore the importance of maintenance therapy in preventing relapses.

Our study has both strengths and limitations. The main limitation is the nonrandomized treatment, which is prone to confounding, especially confounding by indication of treatment regimens. Although we adjusted for relapse rates, it is possible that we missed the beneficial effects of more aggressive therapies because they were administered to those patients with the most severe disease and, therefore, worse outcomes. Another limitation is the possibility of selection bias because of nondifferential loss to follow‐up, especially since follow‐up times varied. Within our cohort, it is noteworthy that some patients had not received any treatment or had received therapy that did not align with current recommendations at the time of initial diagnosis. A potential reason for this is that they were not managed in a tertiary care center either at disease onset or during its early course. Furthermore, recruitment for our cohort ended in 2018, so more recent cases of PACNS are not included. The main strength of our study is that it includes long‐term observation of the world's largest cohort of the overall rare disease PACNS.

### Conclusions

Among patients with PACNS, patient age, mRS at diagnosis, and histologic findings of the necrotizing pattern were significantly associated with worse outcomes. Furthermore, we found that half of the patients had relapses during the long‐term course and that approximately half of the patients had an unfavorable long‐term functional outcome (mRS, 3–6). Our findings further support the treatment of patients with PACNS with cyclophosphamide alone or in combination with steroids to reduce the frequency of relapse.

## Author Contributions

**Anna Lena Fisse:** Conceptualization; data curation; methodology; project administration; writing – original draft; writing – review and editing. **Nadine Bonberg:** Data curation; formal analysis; methodology; software; validation. **Carolin Beuker:** Conceptualization; data curation; methodology; project administration; writing – original draft; writing – review and editing. **Claudia Pfeuffer:** Data curation; formal analysis; methodology. **Andreas Heidenreich:** Formal analysis; methodology; software; visualization. **Christina Krüger:** Data curation; writing – review and editing. **Milani Deb‐Chatterji:** Data curation; writing – review and editing. **Jana Becker:** Data curation; writing – review and editing. **Stefan T. Gerner:** Data curation; writing – review and editing. **Clemens Küpper:** Data curation; writing – review and editing. **Louisa Nitsch:** Data curation; writing – review and editing. **Roxane‐Isabelle Kestner:** Data curation; writing – review and editing. **Lars Udo Krause:** Data curation; writing – review and editing. **Juliane Herm:** Data curation; writing – review and editing. **Alexander Katalinic:** Data curation; formal analysis; supervision; writing – review and editing. **André Karch:** Data curation; formal analysis; software; supervision; validation; writing – review and editing. **Ralf Gold:** Data curation; writing – review and editing. **Heinz Wiendl:** Data curation; writing – review and editing. **Wolf‐Rüdiger Schäbitz:** Data curation; writing – review and editing. **Gabor C. Petzold:** Data curation; writing – review and editing. **Waltraud Pfeilschifter:** Data curation; writing – review and editing. **Marius Ringelstein:** Data curation; writing – review and editing. **Thorsten R. Doeppner:** Data curation; writing – review and editing. **Lars Kellert:** Data curation; writing – review and editing. **Hagen B. Huttner:** Data curation; writing – review and editing. **Markus Kraemer:** Data curation; writing – review and editing. **Tim Magnus:** Data curation; writing – review and editing. **Karl Georg Haeusler:** Data curation; writing – review and editing. **Heike Minnerup:** Conceptualization; data curation; formal analysis; software; supervision; validation; writing – review and editing. **Jens Minnerup:** Conceptualization; data curation; formal analysis; funding acquisition; investigation; methodology; project administration; writing – review and editing.

## Potential Conflicts of Interest

C.B.: has received speaking fees from Chugai Pharma, a subsidiary of Roche involved in the development of biologics used in the study. H.W.: receives honoraria for acting as a member of Scientific Advisory Boards Novartis, a company producing immunosuppressive agents used in the study and has received speaker honoraria and travel support from Roche and acts as a paid consultant for Roche. His research is funded by F. Hoffmann‐La Roche and Roche Pharma, all of which are manufacturers of immunotherapeutic agents that are used in the study. Board fees were received from AstraZeneca, which manufactures immunomodulatory drugs used in the study. M.K.: has received honoraria for teaching activities from Roche Pharma, Chugai Pharma and Novartis, all of which are involved in the development or manufacture of immunotherapeutic agents used in the study. J.M.: has received speaking fees from Chugai Pharma, a subsidiary of Roche involved in the development of biologics used in the study. The remaining authors have nothing to report.

## Data Availability

The data that support the findings of this study are available on request from the corresponding author.
